# Unveiling Species Diversity Within Early-Diverging Fungi from China XIV: Five New Species of Mucorales

**DOI:** 10.3390/jof12060386

**Published:** 2026-05-27

**Authors:** Wen-Xiu Liu, Jing Zhu, Ning Wang, Heng Zhao, Xiao-Yong Liu, Zhi-Dong Zhang

**Affiliations:** 1 Xinjiang Laboratory of Special Environmental Microbiology, Institute of Microbiology, Xinjiang Academy of Agricultural Sciences, Urumqi 830091, China; 2 College of Life Sciences, Shandong Normal University, Jinan 250358, China; 3 CAS Key Laboratory of Forest Ecology and Silviculture, Institute of Applied Ecology, Chinese Academy of Sciences, Shenyang 110016, China

**Keywords:** Mucoromycetes, taxonomy, morphology, molecular phylogeny, fungal diversity

## Abstract

Mucoralean fungi are mostly saprotrophic. During a fungal investigation of soil in Guangdong and Anhui provinces of China, five new species of Mucorales were discovered, namely *Cunninghamella brevispora* sp. nov., *C. geminata* sp. nov., *Mucor chlamydosporiferus* sp. nov., *M. citrinus* sp. nov., and *M. magnisporus* sp. nov. The identification is based on morphological characteristics, as well as molecular phylogenetics of the internal transcribed spacer (ITS), large subunit ribosomal RNA gene (LSU rDNA), translation elongation factor 1-alpha gene (*TEF1α*), and RNA polymerase II largest subunit gene (*RPB1*). *Cunninghamella brevispora* sp. nov. is sister to *C. guizhouensis*, and is distinguished by short sporangiophores. *Cunninghamella geminata* sp. nov. is sister to *C. subclavata*; rhizoids are absent in the former but well-developed in the latter. *Mucor chlamydosporiferus* sp. nov. is closely related to *M. prayagensis*, and is characterized by abundant chlamydospores. *Mucor citrinus* sp. nov. is closely related to *M. paraorantomantidis*, and is differentiated by pale yellow sporangiospores. *Mucor magnisporus* sp. nov. is sister to *M. merdicola*, and is discriminated by large sporangiospores. To date, with the addition of the five new species described herein, the total number of accepted species in the genus *Cunninghamella* and *Mucor* has increased to 49 and 163, respectively.

## 1. Introduction

The Mucorales is an ancient and evolutionarily important group of early-diverging fungi. Taxonomically, this order includes 16 families, 64 genera, and approximately 499 described species (https://www.catalogueoflife.org/data/taxon/623CT, accessed 14 April 2026) [1]. They are widely distributed worldwide and mainly saprotrophic decomposers in terrestrial ecosystems, playing a key role in carbon and mineral cycling [2,3,4,5]. In addition, this group has important biotechnological value in fields such as food fermentation and enzyme production [6,7,8], while some taxa are opportunistic pathogens causing severe mucormycosis in immunocompromised hosts [9,10,11,12,13,14].

The genus *Cunninghamella* was established by Matruchot in 1903 [15]. A century later, Zheng and Chen [16] conducted a monographic study of this genus mainly based on morphology. Conventional morphological characteristics currently used for species identification in this genus include colony features, sporangiophores, vesicles, and sporangiola [17,18]. Although Liu et al. [19] have integrated morphological observations with molecular phylogenetic analyses (e.g., ITS rDNA and *TEF1α* sequences) and established a robust and widely accepted taxonomic framework, the true species diversity of this genus still lacks in-depth investigation and a clear understanding. Since then, numerous new species of *Cunninghamella* have been described based on phylogenetic data and morphological characteristics [16,20,21,22,23,24]. To date, 75 scientific names under this genus have been recorded (https://www.indexfungorum.org/, accessed 14 April 2026), of which 67 are legitimate, and 47 are accepted (https://www.speciesfungorum.org/names/Names.asp, accessed 14 April 2026).

*Mucor* is the most species-rich genus in the order Mucorales [25]. The genus was first formally described by Fresenius in 1850 [26]. Hitherto, 780 scientific names under this genus have been recorded (https://www.indexfungorum.org/, accessed 14 April 2026), of which 394 are legitimate, and 160 are accepted (https://www.speciesfungorum.org/names/Names.asp, accessed 14 April 2026). Species of *Mucor* have a worldwide distribution and occur mainly in substrates such as soil and animal dung, with most being saprotrophic [27,28,29,30,31,32,33]. Their typical morphological characteristics include rapidly growing colonies, simple or branched sporangiophores, globose non-apophysate sporangia, and a deliquescent sporangial wall covered with granular ornamentation.

During an investigation of soil fungal diversity in southern China, two and three species were found to be new in the genera *Cunninghamella* and *Mucor*, respectively. They are described and illustrated herein. This paper is the fifteenth report in a series on the diversity of early-diverging fungi in China [34,35,36].

## 2. Materials and Methods

### 2.1. Sample Collection and Strain Isolation

In 2025, soil samples were collected from Guangdong and Anhui provinces, China, in accordance with the protocols described by Liu et al. [37] and Zou et al. [38]. Each sample, weighing approximately 50 g, was placed into a sterile sealed bag, which was labeled with essential information including sample number, vegetation type, collection date, longitude, latitude, and altitude. All collected samples were transported to the laboratory and stored in a 4 °C refrigerator (Model: LSC-650E, manufactured by Zhejiang Xingxing Cold Chain Integration Co., Ltd., Taizhou, China).

Fungal isolation and purification were carried out by combining the single-spore isolation technique and the serial dilution plate method [38,39]. Detailed steps are presented below: About 1 g of soil sample was weighed and transferred into a test tube containing 9 mL of sterile water; After thorough shaking, a 10^−1^ soil suspension was prepared; approximately 1 mL of this initial suspension was pipetted into another test tube with 9 mL of sterile water and mixed thoroughly to yield a 10^−2^ suspension; this serial dilution process was repeated successively to prepare suspensions with concentrations of 10^−3^ and 10^−4^.

A 200 μL aliquot of both the 10^−3^ and 10^−4^ dilutions was separately dispensed onto the surface of Rose Bengal Chloramphenicol Agar (RBC) plates (Model: HB0237-3, Qingdao Hope Bio-Technology Co., Ltd., Qingdao, China) [40], which were supplemented with 0.03% streptomycin sulfate. The composition of the RBC medium was as follows: 5.00 g/L peptone, 10.00 g/L glucose, 0.50 g/L MgSO_4_·7H_2_O, 1.00 g/L KH_2_PO_4_, 0.05 g/L rose bengal, 0.10 g/L chloramphenicol, and 15.00 g/L agar. Each inoculated plate was then evenly spread using a sterile glass spreader and incubated in a 25 °C incubator under dark conditions for 3 days.

Subsequently, under a stereomicroscope (Olympus SZX10, Olympus, Tokyo, Japan), sporangia of the target fungal strains were picked using a sterile inoculating loop and transferred onto Potato Dextrose Agar (PDA) plates (Model: HB0233, Qingdao Hope Bio-Technology Co., Ltd., Qingdao, China). The PDA medium consisted of 6.00 g/L potato infusion powder, 20.00 g/L dextrose, and 20.00 g/L agar. After transfer, the plates were incubated in the dark at 25 °C in an incubator (Model: 1010159, Ningbo Jiangnan Instrument Factory, Ningbo, China). Following purification, the isolated strains were preserved in 10% glycerol at 4 °C in a refrigerator (Model: LSC-650E, Zhejiang Xingxing Cold Chain Integration Co., Ltd., Taizhou, China).

The dried type specimens were deposited in the Herbarium Mycologicum Academiae Sinicae (HMAS, Beijing, China). The ex-type cultures were preserved at the China General Microbiological Culture Collection Center (CGMCC, Beijing, China). Duplicate strains were stored at Shandong Normal University (XG, Jinan, China). The taxonomic data for the newly described taxa have been submitted to the Fungal Names database (https://nmdc.cn/fungalnames/, accessed on 15 May 2026).

### 2.2. Morphological Observation

High-definition color digital photography (DP80, Olympus, Tokyo, Japan) was used to record the macroscopic morphological traits of the fungal isolates. Concurrently, the microscopic morphological characteristics were examined with an Olympus stereomicroscope (SZX10, Tokyo, Japan) and an Olympus light microscope (BX53, Tokyo, Japan). Following these observations, Digimizer software (https://www.digimizer.com/, accessed on 7 February 2026) was applied to conduct microstructure measurements, where each morphological characteristic—including sporangiophores, columellae, and sporangiospores—was measured a minimum of 50 times [41]. To finalize the image processing, Adobe Photoshop software (https://www.adobe.com/products/photoshop.html, accessed on 12 February 2026) was adopted for the typesetting of the captured microstructure images.

### 2.3. DNA Extraction, PCR Amplification, and Sequencing

Strains were inoculated onto PDA medium and incubated in a 25 °C incubator under dark conditions for 3 to 5 days. Fungal genomic DNA was extracted using either the Beaver Beads Plant DNA Kit (BEAVER Biomedical Engineering Co., Ltd., Suzhou, China, Cat. No.: 70409-20) or the CTAB method [42,43]. Polymerase chain reaction (PCR) was employed to amplify the following gene regions: the internal transcribed spacer (ITS), the large subunit ribosomal DNA (LSU rDNA), the translation elongation factor 1-alpha (TEF-1α), and the RNA polymerase II largest subunit gene (RPB1). The specific primer pairs used for each gene region were ITS4/ITS5 [44] for ITS, LR0R/LR5 [45] for LSU rDNA, EF1-983F/TEF1LLErev [46,47] for TEF-1α, f1843/R3096 [48] for RPB1, respectively (Table 1).

PCR amplification was performed in a total reaction volume of 25 μL, consisting of 12.5 μL of 2× Hieff Canace^®^ Plus PCR Master Mix (Yeasen Biotechnology, Shanghai, China; Cat. No.: 10154ES03), 9.5 μL of double-distilled water (ddH_2_O), 1 μL each of the forward and reverse primers (10 μM, TsingKe Biotechnology Co., Ltd., Beijing, China), and 1 μL of fungal DNA template. After amplification, the PCR products were separated by 1% agarose gel electrophoresis and visualized under ultraviolet light [49]. Target bands were cut out and purified using a Gel Extraction Kit (Cat. No.: AE0101-C; Shandong Sparkjade Biotechnology Co., Ltd., Jinan, China), followed by DNA sequencing performed by Sangon Biotech (Shanghai) Co., Ltd. (Shanghai, China).

Consensus sequences were assembled using Geneious Prime 2025.0.2 software (https://www.geneious.com, accessed on 30 January 2026). All obtained sequences were deposited in the National Center for Biotechnology Information (https://www.ncbi.nlm.nih.gov/, accessed on 9 March 2026), with the corresponding accession numbers listed in Table S1.

### 2.4. Phylogenetic Analyses

All reference sequences were retrieved from the National Center for Biotechnology Information (https://www.ncbi.nlm.nih.gov/, accessed on 9 March 2026) by referring to recently published studies on the genus *Cunninghamella* and *Mucor* [34,35], and comprehensive details of all sequences are presented in Table S1. Sequence alignment and concatenation were implemented using Geneious Prime 2025.0.2 software. *Cunninghamella* was analyzed based on the combined ITS-LSU-*TEF1α* sequence dataset, while *Mucor* was analyzed based on the combined ITS-LSU-*RPB1* sequence dataset. Phylogenetic trees were constructed using two analytical approaches: maximum likelihood (ML) and Bayesian inference (BI).

The ML analysis was carried out on the CIPRES Science Gateway (https://www.phylo.org/) using RAxML 8.2.4 software, with 1000 bootstrap replicates performed to assess branch support [50]. For the Bayesian inference (BI) analysis, the GTR + I + G model was adopted, with sampling conducted at every 1000 generations. Eight cold Markov chains were run simultaneously for a total of 2,000,000 generations [50,51]. The resulting phylogenetic tree was optimized via the iTOL web platform (https://itol.embl.de, accessed on 10 March 2026) and visually refined using Adobe Illustrator CC 2019 software (https://adobe.com/products/illustrator, accessed on 11 March 2026).

## 3. Results

### 3.1. Phylogeny

**Figure 1:**
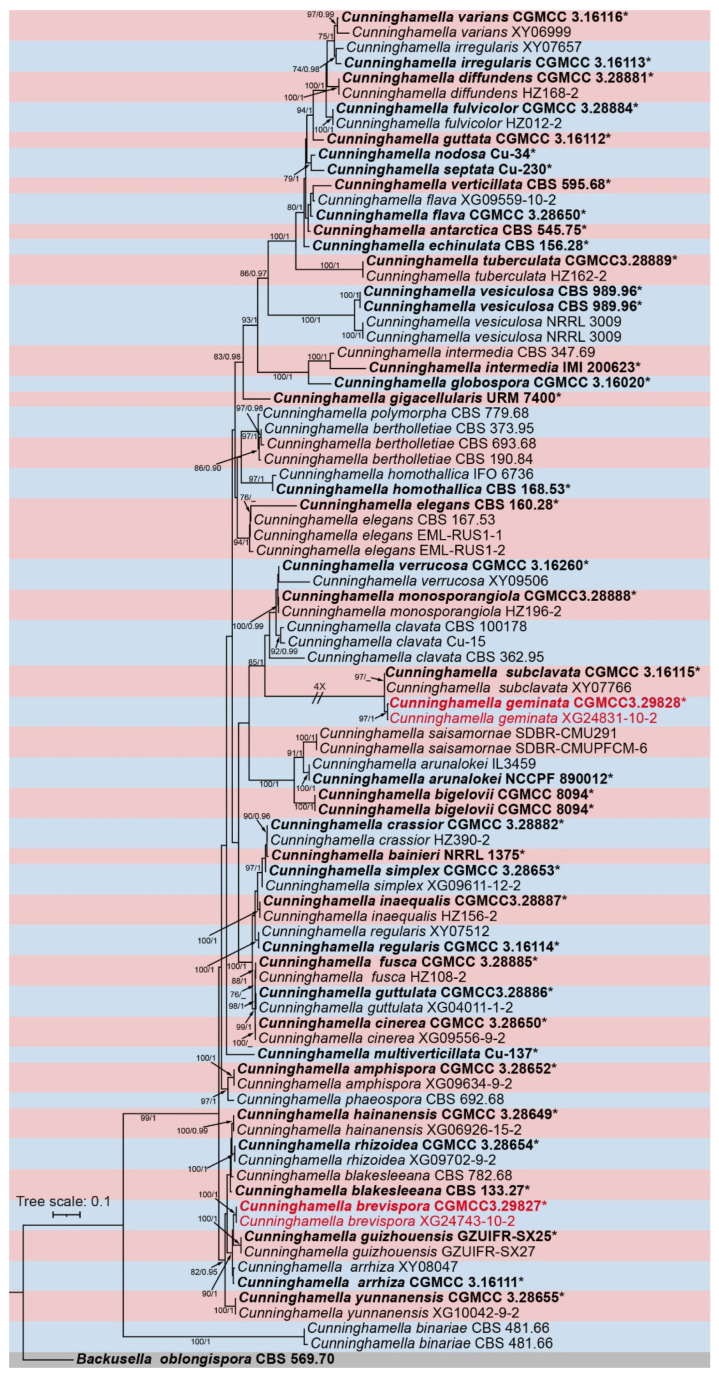
The maximum likelihood (ML) phylogenetic tree of *Cunninghamella* was constructed based on the concatenated sequences of ITS, LSU and *TEF-1α*, with *Backusella oblongispora* as outgroup. The branch robustness is indicated at each node by the maximum likelihood bootstrap values (MLBV ≥ 70%) and Bayesian inference posterior probabilities (BIPP ≥ 0.90), which are separated by a slash “/”. Ex-type or ex-holotype strains are marked in bold black font with an asterisk “*”; the novel species proposed in this study are highlighted in red. Branches that have been shortened are denoted by a double slash “//”, with “×” indicating the shortening fold. The scale bar represents the number of substitutions per site.

The molecular dataset of *Cunninghamella* contains a total of 89 strains, covering 48 species of the genus *Cunninghamella* and the outgroup species *Backusella oblongispora*, with 4245 characters in total. These sites span the ITS ribosomal DNA (1–1465), LSU ribosomal DNA (1466–2855), and the *TEF1α* gene (2856–4245). Among these characters, 1836 are conserved sites, 539 are variable but parsimony-uninformative sites, and 1870 are parsimony-informative sites. The MrModeltest analysis indicated that the GTR + I + G evolutionary model with Dirichlet base frequencies is suitable for all three gene partitions set in the Bayesian inference (BI) analysis. Given that the topological structures of the maximum likelihood (ML) phylogenetic tree and the Bayesian inference tree are consistent, the ML tree was selected as the representative topology for comprehensive display in this study (Figure 1). Notably, the four *Cunninghamella* strains isolated in this study formed two independent and fully supported clades in the phylogenetic tree (Figure 1).

**Figure 2:**
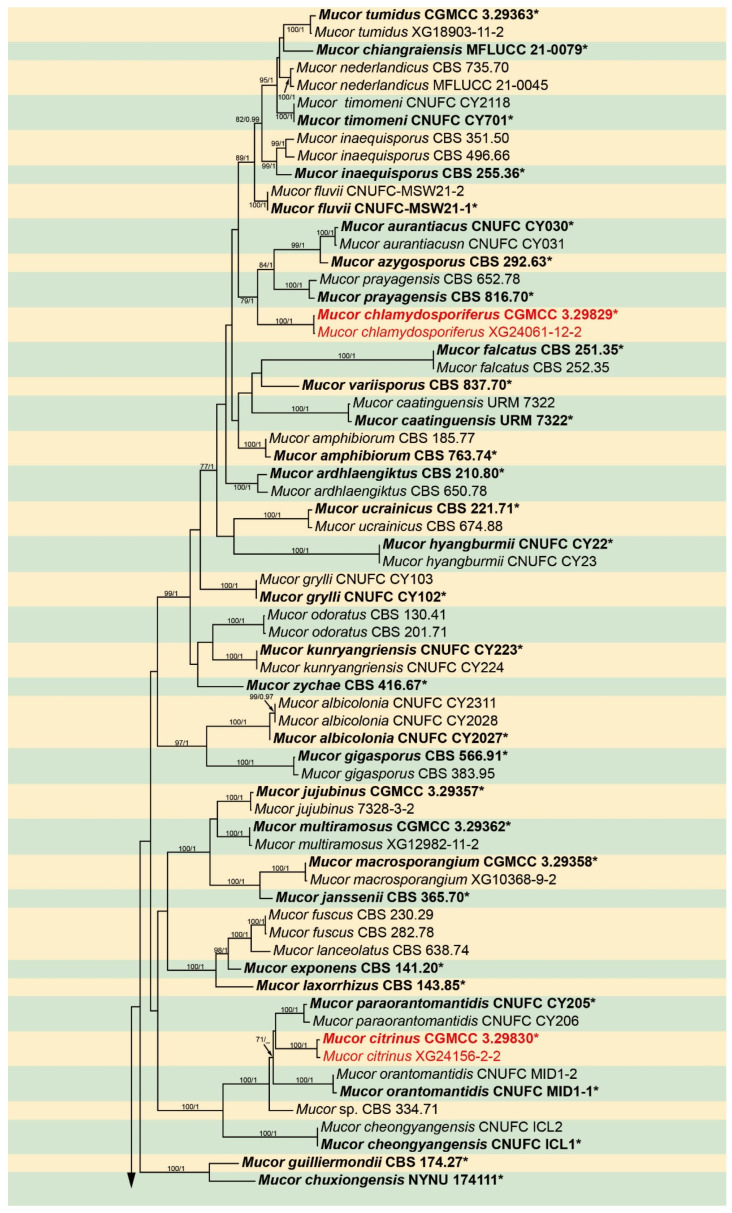

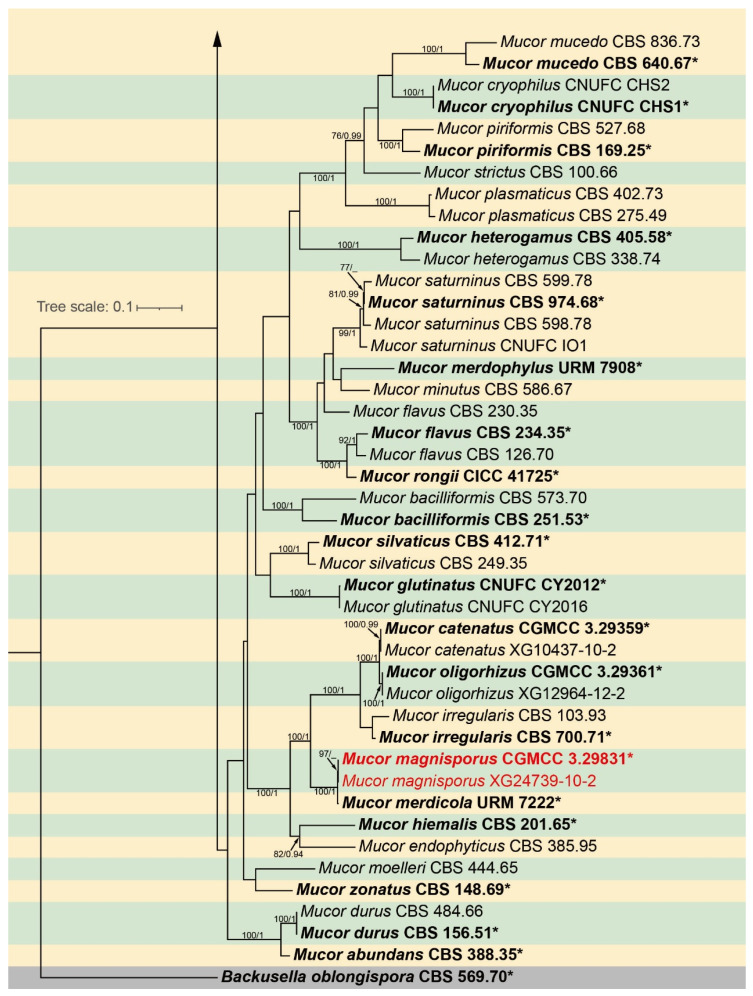
The maximum likelihood (ML) phylogenetic tree of *Mucor* was constructed based on the concatenated sequences of ITS, LSU and RPB1, with *Backusella oblongispora* as outgroup. The branch robustness is indicated at each node by the maximum likelihood bootstrap values (MLBV ≥ 70%) and Bayesian inference posterior probabilities (BIPP ≥ 0.90), which are separated by a slash “/”. Ex-type or ex-holotype strains are marked in bold black font with an asterisk “*”; the novel species isolated in this study are highlighted in red. The scale bar represents the number of substitutions per site. The phylogenetic tree is split into two parts, which are connected by an arrow at the top and bottom.

The molecular dataset of *Mucor* comprises a total of 113 strains, covering 63 species of the genus *Mucor* and the outgroup species *Backusella oblongispora*, with 2911 characteristic sites in total. These sites span the ITS ribosomal DNA (1–1190), LSU ribosomal DNA (1191–1886), and the *RPB1* gene (1887–2911). Among these characteristic sites, 1594 are conserved sites, 231 are variable but parsimony-uninformative sites, and 1086 are parsimony-informative sites. The MrModeltest analysis indicated that the GTR + I + G evolutionary model with Dirichlet base frequencies is suitable for all three gene partitions set in the Bayesian inference (BI) analysis. Given that the topological structures of the maximum likelihood (ML) phylogenetic tree and the Bayesian inference tree are consistent, the ML tree was selected as the representative topology for comprehensive display in this study (Figure 2). Notably, the six *Mucor* strains isolated in this study formed three independent and fully supported clades in the phylogenetic tree (Figure 2).

### 3.2. Taxonomy

**Figure 3:**
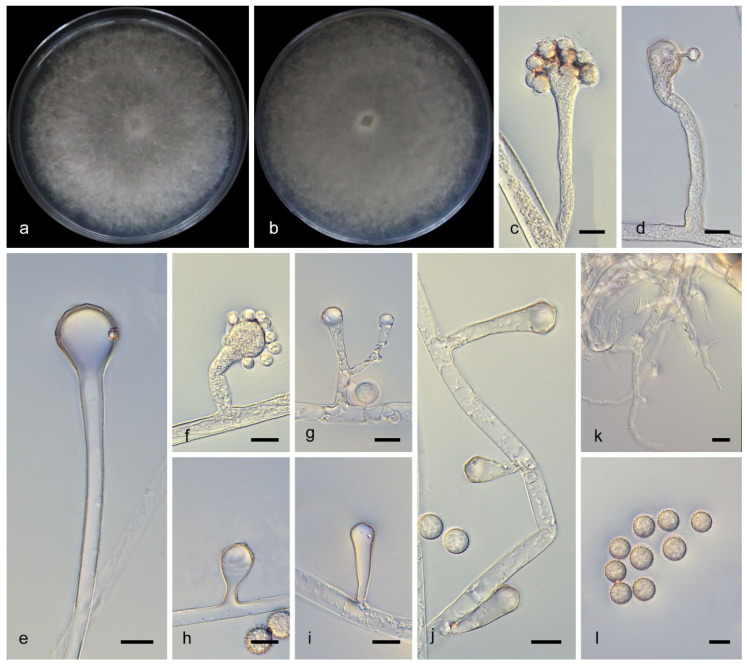
Morphologies of *Cunninghamella brevispora* ex-holotype CGMCC 3.29827. (**a**,**b**) Colonies on PDA, (**a**) obverse, (**b**) reverse; (**c**,**d**,**f**) vesicles with sporangiola; (**e**,**h**–**j**) sporangiophores; (**g**) sporangiophores with branching patterns; (**k**) rhizoids; (**l**) sporangiospores. Scale bars: (**c**–**l**) 10 μm.

*Cunninghamella brevispora* W.X. Liu, J. Zhu and X.Y. Liu, sp. nov., Figure 3.

Fungal Names: FN 5737739.

Type. China, Guangdong Province, Yunfu City, Yun’an District. (22°71′73″ N, 111°93′42″ E, altitude 282.7 m), from soil, 30 December 2025, W.X. Liu, holotype HMAS 354591, ex-holotype living culture CGMCC 3.29827 (=XG24743-10-1).

Etymology: The epithet *brevispora* (Lat.) refers to short sporangiophores.

Description. Colonies on PDA at 25 °C for 6 days reach 80 mm in diameter; they are initially white, gradually becoming pale gray and floccose with age. Hyphae are aseptate when young, septate at maturity, branched, and 3.8–14.4 μm wide. Rhizoids are root-like. Sporangiophores arising from stolons are mostly short, erect or curved, unbranched or simple-branched, and without verticillate branching; the main stalk gradually widened upwards and was 1.7–5.3 μm wide. Vesicles are subspherical with rough walls, 6.5–21.4 μm long and 6.0–18.6 μm wide. Sporangiola are borne on vesicles; they are spherical, echinulate, and 10.2–11.5 μm in diameter. Chlamydospores are absent. Zygospores unknown.

Additional strains examined. China, Guangdong Province, Yunfu City, Yun’an District. (22°71′73″ N, 111°93′42″ E, altitude 282.7 m), from soil, 30 December 2025, W.X. Liu, living culture XG24743-10-2.

Notes. Based on the ITS–LSU–*TEF1α* phylogenetic tree, strains CGMCC 3.29827 and XG24743-10-2 clustered together in a single clade with MLBS/BIPP = 100/1.00 (Figure 1) and formed a sister group with *Cunninghamella guizhouensis* [19]. Morphologically, the sporangiophores of these two strains are mostly short and small, whereas those of *C. guizhouensis* are mostly long and slender. Based on molecular phylogenetic and morphological evidence, these two strains are described as a new species, *Cunninghamella brevispora*.

**Figure 4:**
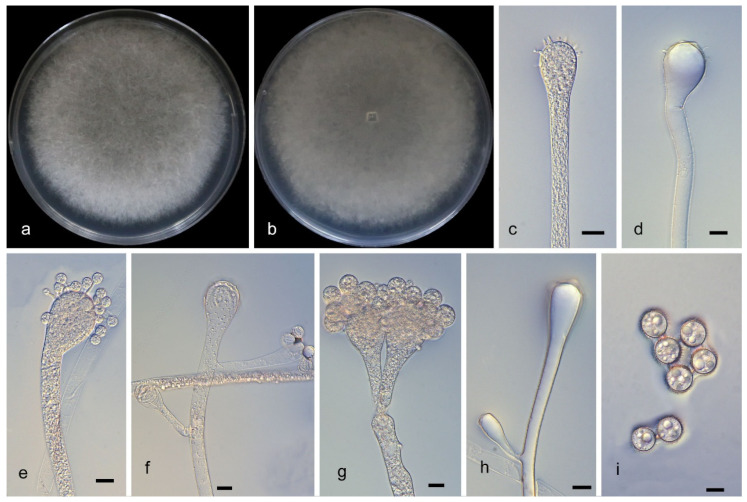
Morphologies of *Cunninghamella geminata* ex-holotype CGMCC 3.29828. (**a**,**b**) Colonies on PDA, (**a**) obverse, (**b**) reverse; (**c**,**d**) sporangiophores; (**e**) vesicles with sporangiola; (**f**–**h**) sporangiophores with branching patterns; (**i**) sporangiola. Scale bars: (**c**–**i**) 10 μm.

*Cunninghamella geminata* W.X. Liu, Zhi Dong Zhang and X.Y. Liu, sp. nov., Figure 4.

Fungal Names: FN 573740.

Type. China, Guangdong Province, Maoming City, Huazhou City, 380 Xiangdao. (22°09′62″ N, 110°44′47″ E, altitude 95.8 m), from soil, 30 December 2025, W.X. Liu, holotype HMAS 354592, ex-holotype living culture CGMCC 3.29828 (=XG24831-10-1).

Etymology: The epithet *geminata* (Lat.) refers to paired sporangia.

Description. Colonies on PDA at 25 °C for 6 days reach 80 mm in diameter; they are initially white, and gradually turn dark gray and floccose with age. Hyphae are aseptate when young, septate at maturity, branched, and 2.6–17.3 μm wide. Rhizoids are absent. Sporangiophores arising from stolons are erect or curved, unbranched or simple-branched, without verticillate branches, gradually widen upwards, and are 12.0–3.8 μm wide. Vesicles are elliptical, with rough walls, 11.6–40.5 μm long and 5.7–30.4 μm wide. Sporangiola borne on vesicles are spherical, echinulate, and 8.5–22.6 μm in diameter. Chlamydospores are absent. Zygospores are not observed.

Additional strains examined. China, Guangdong Province, Maoming City, Huazhou City, 380 Xiangdao. (22°09′62″ N, 110°44′47″ E, altitude 95.8 m), from soil, 30 December 2025, W.X. Liu, living culture XG24831-10-2; China, Guangdong Province, Maoming City, Dianbai District, 641 County Road. (21°83′32″ N, 111°28′41″ E, altitude 108.0 m), from soil, 9 January 2026, W.X. Liu, living culture XG24858-2.

Notes. Based on the ITS–LSU–*TEF1α* phylogenetic tree, strains CGMCC 3.29828 and XG24831-10-2 formed a single clade with MLBS/BIPP = 97/1.00 (Figure 1), sister to *Cunninghamella subclavata* [25]. Morphologically, rhizoids and apophyses were absent in these two strains, whereas *C. subclavata* possesses well-developed rhizoids and distinct apophyses. Based on molecular phylogenetic and morphological evidence, these two strains are described as a new species, *Cunninghamella geminata*.

**Figure 5:**
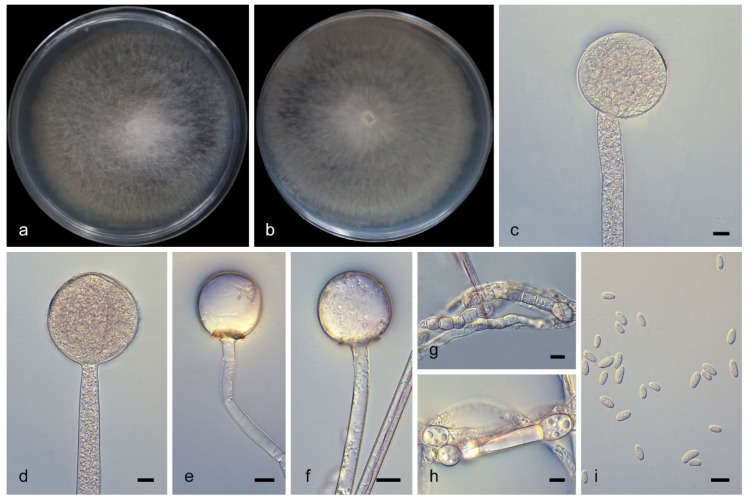
Morphologies of *Mucor chlamydosporiferus* ex-holotype CGMCC 3.29829. (**a**,**b**) Colonies on PDA, (**a**) obverse, (**b**) reverse; (**c**,**d**) sporangia; (**e**,**f**) columellae; (**g**,**h**) chlamydospores; (**i**) sporangiospores. Scale bars: (**c**–**i**) 10 μm.

*Mucor chlamydosporiferus* W.X. Liu, J. Zhu and X.Y. Liu, sp. nov., Figure 5.

Fungal Names: FN 573741.

Type. China, Anhui Province, Lu’an City, Huoshan County. (31°12′96″ N, 116°11′82″ E, altitude 853.3 m), from soil, 25 November 2025, W.X. Liu, holotype HMAS 354593, ex-holotype living culture CGMCC 3.29829 (=XG24061-12-1).

Etymology: The epithet *chlamydosporiferus* (Lat.) refers to abundant chlamydospores.

Description. Colonies on PDA at 25 °C for 9 days reach 80 mm in diameter; they are white at first, becoming pale brown and floccose with age. Hyphae grow radially, are unbranched, hyaline, aseptate when young, septate at maturity, and 3.8–11.8 μm wide. Rhizoids are absent. Sporangiophores arising from both substrate and aerial hyphae are erect or occasionally slightly curved, unbranched, hyaline, slightly pale brown, and 2.5–14.3 μm wide. Sporangia are globose, pale brown, and 48.5–81.8 μm in diameter. Collars are present. Columellae are globose or ovoid, hyaline, smooth-walled, 9.4–49.3 μm long and 10.3–47.7 μm wide. Sporangiospores are fusiform and 5.7–8.7 μm long and 2.7–4.0 μm wide. Chlamydospores produce hyphae in substrate, are globose or ellipsoid, and 6.1–18.1 μm long and 7.7–15.7 μm wide. Zygospores are not observed.

Additional strains examined. China, Anhui Province, Lu’an City, Huoshan County. (31°12′96″ N, 116°11′82″ E, altitude 853.3 m), from soil, 25 November 2025, W.X. Liu, living culture XG24061-12-2; ibid., from soil, 25 November 2025, W.X. Liu, living culture XG24064-11; ibid., from soil, 25 November 2025, W.X. Liu, living culture XG24065-11; ibid., from soil, 25 November 2025, W.X. Liu, living culture XG24067-11; China, Anhui Province, Anqing City, Yuexi County. (31°03′46″ N, 116°11′03″ E, altitude 841.0 m), from soil, 27 November 2025, W.X. Liu, living culture XG24070-13; ibid., from soil, 27 November 2025, W.X. Liu, living culture XG24071-12; China, Hainan Province, Changjiang Li Autonomous County, near the Yaja Scenic Area of Bawangling National Forest Park. (19°04′20″ N, 109°58′70″ E, altitude 563.5 m), from soil, 7 January 2026, W.X. Liu, living culture XG26708-2; China, Hainan Province, Ding’an County, 226 County Road, near Hainan Wenbifeng Daoist Culture Garden–Yuchan Palace. (19°55′34″ N, 110°36′33″ E, altitude 54.4 m), from soil, 20 January 2026, W.X. Liu, living culture XG26810-1; China, Beijing, Chaoyang District, Lin’ao Shopping Center. (40°03′26″ N, 116°40′33″ E, altitude 35.0 m), from feces, 20 January 2026, W.X. Liu, living culture XG 26828-3.

Notes. Based on the ITS–LSU–*RPB1* phylogenetic tree, strains CGMCC 3.29829 and XG24061-12-2 formed a well-supported clade with MLBS/BIPP = 100/1.00 (Figure 1), phylogenetically closely related to *Mucor prayagensis* [52]. Morphologically, abundant chlamydospores were observed in these two strains, whereas no chlamydospores were found in *M. prayagensis*. Molecular phylogenetic and morphological evidence indicate that these two strains represent a new species, being described herein as *Mucor chlamydosporiferus*.

**Figure 6:**
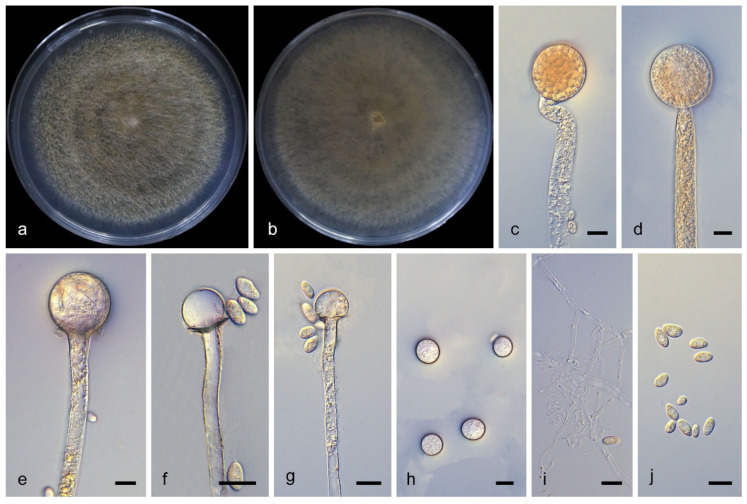
Morphologies of *Mucor citrinus* ex-holotype CGMCC 3.29830. (**a**,**b**) Colonies on PDA, (**a**) obverse, (**b**) reverse; (**c**,**d**) sporangia; (**e**–**g**) columellae; (**h**) chlamydospores; (**i**) rhizoids; (**j**) sporangiospores. Scale bars: (**c**–**j**) 10 μm.

*Mucor citrinus* W.X. Liu, Zhi Dong Zhang, J. Zhu and X.Y. Liu, sp. nov., Figure 6.

Fungal Names: FN 573742.

Type. China, Anhui Province, Huangshan City, Huangshan District, 530 National Road. (30°12′21″ N, 117°98′15″ E, altitude 326.0 m), from soil, 25 November 2025, W.X. Liu, holotype HMAS 354594, ex-holotype living culture CGMCC 3.29830 (=XG24156-2-1).

Etymology: The epithet *citrinus* (Lat.) refers to orange-yellow colonies.

Description. Colonies on PDA at 25 °C for 6 days reach 80 mm in diameter; they are white at first, becoming orange-yellow and floccose with age. Hyphae grow radially, are unbranched, hyaline, aseptate when young, septate at maturity, and 4.3–32.5 μm wide. Rhizoids are present. Sporangiophores arising from both substrate and aerial hyphae are erect or occasionally slightly curved, unbranched, hyaline or slightly yellow-pigmented, and 2.9–19.6 μm wide. Sporangia are globose, orange-yellow, and 26.4–39.6 μm in diameter. Collars are present. Columellae are globose or subglobose, hyaline, smooth-walled, 7.8–28.5 μm long and 7.5–36.7 μm wide. Sporangiospores are fusiform, 4.7–11.3 μm long and 2.7–6.3 μm wide. Chlamydospores are present and globose, 10.1–15.1 μm in diameter. Zygospores are not observed.

Additional strains examined. China, Anhui Province, Huangshan City, Huangshan District, 530 National Road. (30°12′21″ N, 117°98′15″ E, altitude 326.0 m), from soil, 25 November 2025, W.X. Liu, living culture XG24156-2-2.

Notes. Based on the ITS–LSU–*RPB1* phylogenetic tree, strains CGMCC 3.29830 and XG24156-2-2 clustered together to form a single clade with a support value of MLBS/BIPP = 100/1.00 (Figure 1), showing a close phylogenetic relationship with *Mucor paraorantomantidis* [53]. Morphologically, the sporangiospores of these two strains are pale yellow, and their chlamydospores are solitary; in contrast, those of *M. paraorantomantidis* have hyaline to grayish-green sporangiospores and mostly produce chlamydospores in chains. Combining molecular phylogenetic and morphological evidence, these two strains are described as a novel species, *Mucor citrinus*.

**Figure 7:**
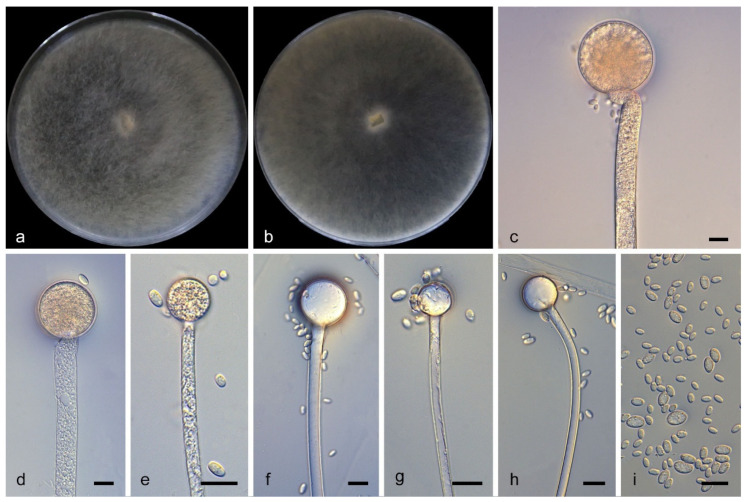
Morphologies of *Mucor magnisporus* ex-holotype CGMCC 3.29831. (**a**,**b**) Colonies on PDA, (**a**) obverse, (**b**) reverse; (**c**–**e**) sporangia; (**f**–**h**) columellae; (**i**) sporangiospores. Scale bars: (**c**–**i**) 10 μm.

*Mucor magnisporus* W.X. Liu, J. Zhu and X.Y. Liu, sp. nov., Figure 7.

Fungal Names: FN 573743.

Type. China, Guangdong Province, Yunfu City, Yun’an District. (22°71′73″ N, 111°93′42″ E, altitude 282.7 m), from soil, 30 December 2025, W.X. Liu, holotype HMAS 354595, ex-holotype living culture CGMCC 3.29831 (=XG24739-10-1).

Etymology: The epithet *magnisporus* (Lat.) refers to large sporangiospores.

Description. Colonies on PDA at 25 °C for 6 days reach 85 mm in diameter; they are white at first, becoming creamy yellow and floccose with age. Hyphae grow radially, are unbranched, hyaline, aseptate when young, septate at maturity, and 3.3–17.8 μm wide. Rhizoids are absent. Sporangiophores arising from both substrate and aerial hyphae are erect or occasionally slightly curved, unbranched, hyaline or slightly pigmented, and 2.6–16.3 μm wide. Sporangia are globose, yellow, and 10.9–46.4 μm in diameter. Collars are absent. Columellae are globose, hyaline, smooth-walled, 7.7–35.7 μm long and 6.3–35.0 μm wide. Sporangiospores are fusiform, relatively large, 3.1–9.6 μm long and 1.6–5.2 μm wide. Chlamydospores are absent. Zygospores unknown.

Additional strains examined. China, Guangdong Province, Yunfu City, Yun’an District. (22°71′73″ N, 111°93′42″ E, altitude 282.7 m), from soil, 30 December 2025, W.X. Liu, living culture XG24739-10-2.

Notes. Based on the ITS–LSU–*RPB1* phylogenetic tree, strains CGMCC 3.29831 and XG24739-10-2 formed a clade with MLBS = 97 (Figure 1), and were sister to *Mucor merdicola* [54]. Morphologically, the sporangiophores of these two strains are unbranched, whereas *M. merdicola* shows repeatedly sympodially branched sporangiophores. Based on combined molecular phylogenetic and morphological evidence, these two strains are described as a new species, *Mucor magnisporus*.

## 4. Discussion

In the multi-locus phylogenetic tree, each of these five new species formed a well-supported clade (Figure 1 and Figure 2). *Cunninghamella brevispora* and *C. guizhouensis* [19] are sister taxa. Compared with *C. guizhouensis*, the sporangiophores of *C. brevispora* are much shorter, whereas those of *C. guizhouensis* are mostly long and slender. *Cunninghamella geminata* and *C. subclavata* [25] are sister taxa. However, rhizoids and apophyses are absent in *C. geminata*, while *C. subclavata* possesses well-developed rhizoids and distinct apophyses. *Mucor chlamydosporiferus* is phylogenetically closely related to *M. prayagensis* [52]. Compared with *M. prayagensis*, abundant chlamydospores were observed in *M. chlamydosporiferus*, whereas no chlamydospores were detected in *M. prayagensis*. *Mucor citrinus* is closely related to *M. paraorantomantidis* [53]. In contrast, the sporangiospores of *M. citrinus* are pale yellow, and its chlamydospores are mostly solitary, whereas those of *M. paraorantomantidis* have grayish-green sporangiospores as well as chlamydospores in chains. *Mucor magnisporus* and *M. merdicola* [54] are sister taxa. The sporangiophores of *M. magnisporus* are unbranched, while those of *M. merdicola* show repeatedly sympodial branching.

Mucorales is the largest group in Mucoromycetes, characterized by rapid growth, coenocytic hyphae, and typical sporangial structures [53,55,56]. Fungi of the order have a worldwide distribution, with the highest diversity in temperate, subtropical, and tropical regions [57,58,59]. Most species in this group are saprotrophic, acting as key decomposers of organic matter in nature. They mainly inhabit soil, decaying plants, animal dung, air, and food substrates, with soil being their common habitat [60,61,62]. New species of Mucorales are continuously being discovered and described globally, especially from China [18,19,25,34,35]. In this study, five new species isolated from Guangdong and Anhui provinces of China are described based on morphological characteristics and phylogenetic analyses. These findings further enrich the species diversity of Mucorales and improve our understanding of the structural characteristics, distribution range, and ecological preferences of mucoralean fungi.

## Figures and Tables

**Table 1:** PCR primers and amplification programs employed in this study.

Loci	PCR Primers	Primer Sequence (5′–3′)	PCR Cycles	References
ITS	ITS5	GGA AGT AAA AGT CGT AAC AAG G	95 °C 5 min; (95 °C 30 s, 55 °C 30 s, 72 °C 1 min) × 35 cycles; 72 °C 10 min	[44]
	ITS4	TCC TCC GCT TAT TGA TAT GC		
LSU	LR0R	GTA CCC GCT GAA CTT AAG C	95 °C 5 min; (95 °C 50 s, 47 °C 30 s, 72 °C 1.5 min) × 35 cycles; 72 °C 10 min	[45]
	LR5	TCC TGA GGG AAA CTT CG		
*TEF1α*	EF1-983F	GCYCCYGGHCAYCGTGAYTTYAT	95 °C 5 min; (95 °C: 30 s, 55 °C 60 s, 72 °C: 60 s) × 30 cycles; 72 °C 10 min	[46,47]
	TEF1LLErev	AAC TTG CAG GCA ATG TGG		
*RPB1*	F1843	ATT TYG AYG GTG AYG ARA TGA AC	94 °C 30 s; (51 °C 30 s, 72 °C 1 min) × 5 cycles; (49 °C 30 s, 72 °C 1 min) × 5 cycles; (47 °C 30 s, 72 °C 1 min) × 5 cycles; 72 °C 10 min	[48]
	R3096	GRA CRG TDC CRT CAT AYT TRA CC		

## Data Availability

All sequences were uploaded to the GenBank database; the accession numbers are provided in Tables S1 and S2. The original contributions presented in this study are included in the article and Supplementary Material. Further inquiries can be directed to the corresponding author.

## Supplementary Materials

The following supporting information can be downloaded at: https://www.mdpi.com/article/10.3390/jof12060386/s1, Table S1: GenBank accession numbers of *Cunninghamella* and *Backusella* strains in this study; Table S2: GenBank accession numbers of *Mucor* and *Backusella* strains in this study.
