# Supplementary material for: Unveiling Species Diversity Within Early-Diverging Fungi from China XIV: Five New Species of Mucorales
Source: J Fungi (Basel). 2026 May 27;12(6):386. doi: 10.3390/jof12060386 (PMC13302190; doi:10.3390/jof12060386)
Supplement: Supplementary file 1 [file jof-12-00386-s001.zip › Table S1.pdf]

Table S1. GenBank accession numbers of *Cunninghamella* and *Backusella* strains in this study.

**Species**	**Strains**	**GenBank Accession Numbers**		
		**ITS**	**LSU**	***TEF1α***
C. amphispora	CGMCC 3.28652*	PV089203	PV123104	PV200769
	XG09634-9-2	PV089204	PV123105	PV200770
C. antarctica	CBS 545.75 T	JN205893	JN206597	KJ156492
C. arrhiza	CGMCC 3.16111 T	OL678142	PQ399916	NA
	XY08047	OL678143	NA	NA
C. arunalokei	IL3459	MN431159	MN431158	NA
	NCCPF 890012 T	NR_177485	NG153887	NA
C. bainieri	CBS 481.66	MH858865	MH870507	KJ156495
	CGMCC 8094 T	KJ013403	KJ013405	KJ395944
	NRRL 1375 T	AF254935	NA	NA
C. bertholletiae	CBS 190.84	JN205878	HM849701	NA
	CBS 373.95	JN205873	NA	KJ156497
	CBS 693.68	AF254931	MH870924	KJ156490
C. bigelovii	CGMCC 8094 T	KJ013403	KJ013405	KJ395944
C. binariae	CBS 481.66	MH858865	MH870507	KJ156495
C. blakesleeana	CBS 133.27 T	NR119974	MH866397	KJ156479
	CBS 782.68	JN205869	MH870950	KJ156478
C. brevispora	**CGMCC3.29827 T**	**PZ272631**	**PZ278609**	**NA**
	**XG24743-10-2**	**PZ272630**	**PZ278610**	**NA**
C. cinerea	CGMCC 3.28650*	PV089197	PV123098	PV172612
	XG09556-9-2	PV089198	PV123099	PV172613
C. clavata	CBS 100178	JN205890	JN206604	KJ156477
	Cu-15	AF254942	NA	NA
	CBS 362.95	JN205891	NA	NA
C. crassior	CGMCC3.28882 T	PV239680	PV235924	PV254891
	HZ390-2	PV239681	PV235925	PV254892
C. diffundens	CGMCC3.28881 T	PV239676	PV235920	PV254889
	HZ168-2	PV239677	PV235921	PV254890
C. echinulata	CBS 156.28 T	JN205895	JN939199.1	KJ156500
C. elegans	CBS 160.28 T	AF254928	NR_154747	KJ156470
	CBS 167.53	JN205882	HM849700	KJ156494
	EML-RUS1-1	MF806023	MF806027	NA
	EML-RUS1-2	MF806021	MF806028	NA
C. flava	CGMCC 3.28651 T	PV089199	PV123100	PV200765
	XG09559-10-2	PV089200	PV123101	PV200766
C. fulvicolor	CGMCC3.28884 T	PV239666	PV235910	PV254879
	HZ012-2	PV239667	PV235911	PV254880
C. fusca	CGMCC3.28885 T	PV239664	PV235908	PV254877
	HZ108-2	PV239665	PV235909	PV254878
C. geminata	**CGMCC3.29828 T**	**PZ272633**	**PZ278611**	**PZ291126**
	**XG24831-10-2**	**PZ272634**	**PZ278612**	**PZ291127**
C. globospora	CGMCC 3.16020 T	MW264073	MW264132	NA
C. gigacellularis	URM 7400 T	NR_168760	NG_068773	NA
C. guizhouensis	GZUIFR-SX25 T	MN908596	MN908599	MN912633
	GZUIFR-SX27	MN908598	MN908601	MN912635
C. guttata	CGMCC 3.16112 T	OL678144	PQ399917	NA

C. guttulata	CGMCC3.28886 T	PV235930	PV239686	PV254893
	XG04011-1-2	PV235931	PV239687	PV254894
C. hainanensis	CGMCC 3.28649*	PV089195	PV123096	PV172610
	XG06926-15-2	PV089196	PV123097	PV172611
C. homothallica	CBS 168.53 T	JN205863	JN206605	KJ156498
	IFO 6736	AF254941	NA	NA
C. inaequalis	CGMCC3.28887 T	PV239670	PV235914	PV254883
	HZ156-2	PV239671	PV235915	PV254884
C. intermedia	CBS 347.69	JN205892	JN206606	NA
	IMI 200623 T	AF254939	NA	NA
C. irregularis	CGMCC 3.16113 T	OL678145	PQ399918	NA
	XY07657	OL678146	NA	NA
C. monosporangiola	CGMCC3.28888 T	PV239678	PV235922	PV785980
	HZ196-2	PV239679	PV235923	PV785981
C. multiverticillata	CBS 989.96 T	JN205897	HM849693	KJ156474
	Cu-137 T	AF254933	NA	NA
	NRRL 3009	AF254943	NA	NA
C. nodosa	Cu-34 T	AF346407	NA	NA
C. phaeospora	CBS 692.68 T	JN205864	HM849697	NA
C. polymorpha	CBS 779.68	JN205874	JN206599	NA
C. regularis	CGMCC 3.16114 T	OL678148	PQ399919	NA
	XY07512	OL678150	NA	NA
C. rhizoidea	CGMCC 3.28654	PV089205	PV123106	PV222157
	XG09702-9-2	PV089206	PV123107	PV222158
C. saisamornae	SDBR-CMUPFCM-6	MW709394	MW699571	MW715866
	SDBR-CMU291	MG571234	MW699591	MW715865
C. septata	Cu-230 T	AF346408	NA	NA
C. simplex	CGMCC 3.28653	PV089201	PV123102	PV200767
	XG09611-12-2	PV089202	PV123103	PV200768
C. subclavata	CGMCC 3.16115 T	OL678152	NA	NA
	XY07766	OL678153	NA	NA
C. tuberculata	CGMCC3.28889 T	PV235918	PV239674	PV254887
	HZ162-2	PV235919	PV239675	PV254888
C. varians	CGMCC 3.16116 T	OL678154	PQ399920	NA
	XY06999	OL678155	NA	NA
C. vesiculosa	CBS 989.96 T	JN205897	HM849693	KJ156474
	NRRL 3009	AF254943	NA	NA
C. verrucosa	CGMCC 3.16260	ON262555	ON261192	NA
	XY09506	ON262556	ON261193	NA
C. verticillata	CBS595.68 T	AF254937	NA	NA
C. yunnanensis	CGMCC 3.28655*	PV089207	PV123108	PV222159
	XG10042-9-2	PV089208	PV123109	PV222160
Backusella oblongispora	CBS 569.70 T	JN206251	JN206407	NA

Notes: New species discovered herein are shown in bold. The asterisk "T" indicates the ex-type or ex-holotype strains. The "NA" stands for "not available".
